# Professor P. C. Gaillard, M.D.

**Published:** 1859-03

**Authors:** 


### NECROLOGICAL NOTICES.

PROFESSOR P. C. GAILLARD, M.D., of Charleston, South Carolina.—We record with profound sorrow, the melancholy and premature death of Dr. Peter Cordes Gaillard, a gentleman well known and greatly respected by the members of our profession in this city, and, indeed, throughout our country generally. It was but in our number for last July that we announced his appointment to the Chair of Institutes and Practice of Physic in the Medical College of the State of South Carolina, filling the vacancy made by the removal of Professor Dickson to the Jefferson College. Great hopes were entertained from his elevation to this responsible post, which he was permitted to hold for so brief a period. For a considerable time past he had been struggling with pulmonary disease, which seemed at least to have suspended its progress. But the grief of losing an infant daughter during the fatigues and annoyances of the last sickly summer and autumn, and the recent death of a near connection and dear friend, proved too much for his powers of resistance and endurance, and of late he sunk rapidly, and died after but a few days' confinement to his house. It was indeed a very little while since he persisted, with manly fortitude, in the performance of his numerous weighty and responsible duties.

Dr. Gaillard was one of the founders and first editors of the *Charleston Medical and Surgical Journal*, which he aided to establish upon a sound and lofty basis, and to which he was always a most valued contributor. He was a few years ago President of the Medical Society of South Carolina, and occupied, in succession, every place of honor and trust to which he would permit himself to be nominated, in all the professional associations of his native State. His addresses, as orator, were always well received and highly lauded. His clinical lectures at the hospital and almshouse are still remembered by the pupils who enjoyed the advantage of attendance upon them, as exceedingly interesting and instructive.

At the time of his decease he was one of the Trustees of the Roper Hospital, of which he had previously been attendant and consulting physician. He was also, from the commencement, Treasurer of the Widows' and Orphans' Society, and had been distinguished for his earnest zeal and faithful enthusiasm in promoting this charity, so important to the unfortunate and afflicted among physicians.

Professor Gaillard commenced his winter course in November, 1858, with the most gratifying anticipations of success and advancement. Already deservedly popular, both as teacher and practitioner, his introductory address gave much satisfaction, and succeeding lectures only tended to confirm every favorable expectation. But, alas! all these hopes are blighted, all these prospects ended by his lamentable death!

In this event our profession has lost one of its strongest pillars; one of its brightest ornaments; a diligent inquirer after truth; a clear thinker; a model gentleman. Gifted by nature with an engaging presence and a fine intellectual capacity, he cultivated this latter with the most conscientious assiduity, while he attracted the love and admiration of all who knew him, by his polished refinement and undeviating courtesy of manner.

Early annoyed by uncertain health, he had successfully struggled with all the difficulties that impeded his outset in life. He had entered upon an extensive and lucrative practice, among the most intelligent and respectable classes of the community, commanding the confidence and winning the universal affection of his patients. He rose steadily in the estimation of his brethren everywhere, and was doubtless destined, had he survived, to fill a large space in the public eye. His election to the Chair of Practice by the unanimous vote of such a body of men as constitute the Board of Trustees of the Medical College of the State of South Carolina, was a bright earnest of his future progress. His native city could have selected no one better fitted to uphold her character for talent and integrity.

And now, all is over, and the grave has closed darkly upon his aspirations after well-merited fame and a career of protracted usefulness and honor. But his memory will ever be green in the hearts of his friends; and while men regard uprightness, truth, intelligence, refinement, and piety, as worthy of esteem and reverence, the name of Gaillard shall not be permitted to fall into oblivion. D.

